# Fertility-Preserving Surgery in a Young Nulligravid Woman with Bilateral Coexistence of a Granulosa Cell Tumor with a Teratoma

**DOI:** 10.1155/2023/9438575

**Published:** 2023-09-21

**Authors:** Pham Ba Nha, Pham Van Tuyen, Nguyen Viet Ha, Nguyen Thi Thu Phuong

**Affiliations:** ^1^Department of Obstetrics and Gynecology, Hanoi Medical University, Hanoi, Vietnam; ^2^Pathology and Cytology Center, Bach Mai Hospital, Hanoi, Vietnam; ^3^Department of Obstetrics and Gynecology, Bach Mai Hospital, Hanoi, Vietnam

## Abstract

**Background:**

The coexistence of a granulosa cell tumor with a teratoma is extremely rare and impossible to diagnose preoperatively. For most patients with advanced age and stage, the standard treatment is hysterectomy and bilateral salpingo-oophorectomy; however, fertility-preserving surgery should be considered for young nulligravid women.

**Case:**

We present a case of a 24-year-old nulligravid female with bilateral adnexal masses, imaging findings of ovarian teratomas, and normal levels of tumor markers. A laparotomy revealed bilateral dermoid cysts, and solid tissue invaded most of the remaining ovarian parenchyma with no signs of malignancy in the uterus and peritoneum space. Consequently, a bilateral oophorectomy was performed to preserve her fertility. Histopathology examination showed mature cystic teratomas coexisting with granulosa cell tumors on both ovaries. Within six months, there were no signs of recurrence on ultrasonography and tumor makers. Combined oral contraceptive pills were prescribed as hormone replacement therapy.

**Conclusion:**

Fertility-preserving surgery can be performed in young women with an ovarian granulosa cell tumor coexisting with a teratoma. Long-term examination, hormone replacement therapy, and in vitro fertilization are required.

## 1. Introduction

The coexistence of a granulosa cell tumor with a teratoma is extremely rare, with only nine cases reported in the literature [1]. The incidence of ovarian granulosa cell tumors and mature cystic teratomas is 0.6-0.8 and 1.4-14.2 per 100,000, respectively [2, 3]. The incidence of having both types is unknown due to the rarity of case reports.

The etiology of this mixed-type tumor is unknown because of the different origins of each type (germ cells for mature teratomas and ovarian mesenchyme and sex cords for granulosa cells). Mature teratomas are benign tumors; however, granulosa cell tumors are low-grade malignancies. The standard treatment of a granulosa cell tumor is hysterectomy and bilateral salpingo-oophorectomy, performed in most reported cases [1, 4, 5]. On the other hand, this surgery may result in severe consequences regarding reproductivity for young women, and a different treatment should be considered.

Here, we present the first young patient with a coexistence of a granulosa cell tumor with a teratoma, who underwent fertility-preserving surgery combined with hormone replacement therapy.

## 2. Case Presentation

A 24-year-old nulligravid woman was hospitalized during an emergency department visit due to a three-month history of persistent lower abdominal pain. Clinical examination revealed two palpable and firm masses on both adnexa, with a diameter of approximately 6-8 cm. A computed tomography scan, with Iohexol contrast, revealed two cystic, fatty, and nonenhancing lesions in the right and left ovary with the size of 86 mm × 55 mm × 51 mm and 71 mm × 40 mm × 34 mm, respectively. No abdominal fluid was noted. Blood work did not detect abnormal levels of CA 125, HE4, and alpha-fetoprotein. Therefore, she was scheduled for ovarian cystectomy due to bilateral cystic teratomas.

**Figure 1:**
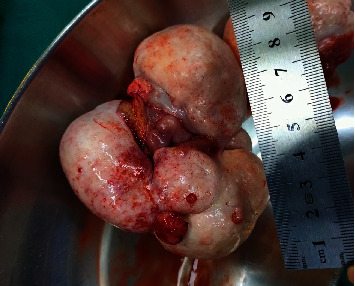
Morphology of the right ovary. The tumor includes 3 thick, firm lobes invading most of the ovary (photo by author).

**Figure 2:**
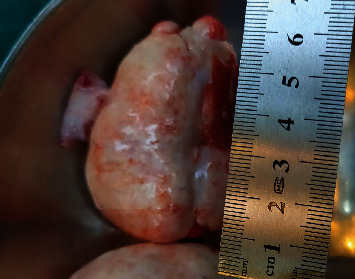
Morphology of the left ovary; a 6 cm x 3 cm firm tumor inside the ovary (photo by author).

**Figure 3:**
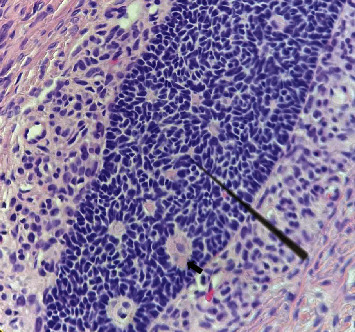
Microscopic histopathology of the granulosa cell tumor. Small polygonal cells are seen with grooved nuclei. Call-Exner bodies filled with eosinophilic material can be seen (black arrow) (photo by author).

During the surgery, 20 milliliters of abdominal fluid were collected, and no suspected metastasis was found in the peritoneal cavity. A 9 cm × 8 cm mass on the right ovary (Figure 1) and a 6 cm × 3 cm mass on the left ovary (Figure 2) were found, which comprised cysts of hair, tufts, and sebaceous materials, along with solid tissue that occupied most of the remaining ovarian parenchyma. No abnormalities of the uterus or fallopian tubes were detected. As the lesions were limited in the ovaries and the patient was young and nulligravid, a bilateral oophorectomy was performed to preserve her fertility. The findings on histopathology and immunochemistry of Synap, Chromo, Inhibin, and Calretinin were consistent with the diagnosis of mature cystic teratomas coexisting with granulosa cell tumors on both ovaries (Figure 3). No malignant cells were found in the abdominal fluid.

The patient returned for a follow-up six months after the operation with no sign of recurrence detected by clinical examination and ultrasonography. The serum levels of CA-125, HE4, and alpha-fetoprotein were within normal ranges. The patient was prescribed combined oral contraceptive pills (0.03 mg ethinylestradiol and 0.15 mg desogestrel) in 21-day cycles as hormone replacement therapy.

## 3. Discussion

To our best knowledge, this is the first case in Vietnam of the coexistence of a granulosa cell tumor and a teratoma in both ovaries for a nulligravid woman of reproductive age. A granulosa cell tumor can exist in the same ovary with a mature cystic teratoma [1, 4–7] or in the contralateral ovary [8]. As such, the bilateral ovarian tumor makes radical surgery inappropriate regarding reproductivity, especially in a young nulligravid woman. The patient was treated with fertility-preserving surgery. After consideration, this surgery can be the best management method regarding socially challenging factors.

Our patient was younger than most reported 40- to 80-year-old cases [1, 4, 5, 9]. It has been noted that the incidence of ovarian granulosa cell tumors is lowest in ages 20-24 and highest in ages 50-64 [2].

While the preoperative clinical examination, computed tomography scan, and tumor makers did not reveal malignant signs, the decisive diagnosis could only be determined by histopathology after the operation. During the surgery, we found bilateral ovarian tumors' morphology and density different from typical ovarian teratomas. Besides cysts with hair, tufts, and sebaceous fluid, the remaining ovarian parenchyma was solid, unlike normal tissue. No signs of ovarian malignancy were detected. Considering the patient's age, gravidity, tumor morphology, and absence of metastases (stage IB) (Table 1), bilateral oophorectomy was performed to preserve her reproductivity.

Postoperative management is expected to maintain reproductivity and monitor recurrence. Hormone replacement therapy and in vitro fertilization are considered suitable methods. Hormone replacement therapy was combined with oral contraceptive pills based on staging without evidence of recurrence [12]. In vitro fertilization with donor oocytes can be applied when the patient plans to be pregnant. In addition, a long-term examination is required with routine clinical follow-ups and serial tumor markers evaluated. No recurrence of this mixed-type tumor within two years [9] is reported. It has been noted that granulosa cell tumors usually recur within five years following initial diagnosis, with the mean surgery-to-recurrence interval being 48-57 months [13].

There are some limitations in our study. The duration of the postoperative examination is six months. The patient needs routine follow-up to evaluate the effects of combined oral contraceptive pills and detect tumor recurrences.

## 4. Conclusion

The coexistence of a granulosa cell tumor with a teratoma is extremely rare. This tumor can develop in both ovaries of young women. In a nulligravid patient of reproductive age, fertility-preserving surgery can be performed in case of no signs of metastases. Postoperative management requires maintaining reproductivity and long-term surveillance. Combined oral contraceptive pills can be prescribed to the patient at an early stage. In vitro fertilization with donor oocytes should be counseled for the pregnancy expectation.

## Figures and Tables

**Table 1:** FIGO staging classification for cancer of the ovary, fallopian tube, and peritoneum [10, 11].

*Stage I: tumor confined to ovaries or fallopian tube(s)*
IA: tumor limited to 1 ovary (capsule intact) or fallopian tube; no tumor on ovarian or fallopian tube surface; no malignant cells in the ascites or peritoneal washings
IB: tumor limited to both ovaries (capsules intact) or fallopian tubes. No tumor on ovarian or fallopian tube surface. No malignant cells in the ascites or peritoneal washings
IC: tumor limited to one or both ovaries or fallopian tubes, with any of the following: IC1: surgical spill IC2: capsule ruptured before surgery or tumor on ovarian or fallopian tube surface IC3: malignant cells in the ascites or peritoneal washings

*Stage II: tumor involves one or both ovaries or fallopian tubes with pelvic extension (below pelvic brim) or peritoneal cancer*
IIA: extension and/or implants on the uterus and/or fallopian tubes/and/or ovaries
IIB: extension to other pelvic intraperitoneal tissues

*Stage III: tumor involves one or both ovaries, or fallopian tubes, or primary peritoneal cancer, with cytologically or histologically confirmed spread to the peritoneum outside the pelvis and/or metastasis to the retroperitoneal lymph nodes*
IIIA1: positive retroperitoneal lymph nodes only (cytologically or histologically proven): IIIA1(i): metastasis up to 10 mm in greatest dimension IIIA1(ii): metastasis more than 10 mm in greatest dimension
IIIA2: microscopic extrapelvic (above the pelvic brim) peritoneal involvement with or without positive retroperitoneal lymph nodes
IIIB: macroscopic peritoneal metastasis beyond the pelvis up to 2 cm in greatest dimension, with or without metastasis to the retroperitoneal lymph nodes
IIIC: Macroscopic peritoneal metastasis beyond the pelvis more than 2 cm in greatest dimension, with or without metastasis to the retroperitoneal lymph nodes (includes extension of tumor to capsule of liver and spleen without parenchymal involvement of either organ)

*Stage IV: distant metastasis excluding peritoneal metastases*
IV A: pleural effusion with positive cytology IV B: metastases to extra-abdominal organs (including inguinal lymph nodes and lymph nodes outside of abdominal cavity)

FIGO: International Federation of Gynecology and Obstetrics.

## Data Availability

Data used to write this case report is accessible through contact with the corresponding author, Pham Ba Nha (email: bnpham2018@gmail.com).
